# Chemical and Physical Methods to Analyze a Multicomponent Traditional Chinese Herbal Prescription Using LC-MS/MS, Electron Microscope, and Congo Red Staining

**DOI:** 10.1155/2013/952796

**Published:** 2013-08-12

**Authors:** Chia-Ming Lu, Mei-Ling Hou, Lie-Chwen Lin, Tung-Hu Tsai

**Affiliations:** ^1^Institute of Traditional Medicine, National Yang-Ming University, Taipei 112, Taiwan; ^2^National Research Institute of Chinese Medicine, Taipei 112, Taiwan; ^3^Graduate Institute of Acupuncture Science, China Medical University, Taichung 404, Taiwan; ^4^Department of Education and Research, Taipei City Hospital, Taipei 103, Taiwan

## Abstract

This study develops several chemical and physical methods to evaluate the quality of a traditional Chinese formulation, Jia-Wei-Xiao-Yao-San. Liquid chromatography-tandem mass spectrometry (LC-MS/MS) coupled with electrospray ionization was used to measure the herbal biomarkers of saikosaponin A, saikosaponin D, ferulic acid, and paeoniflorin from this herbal formula. A scanning electron microscope (SEM) and light microscopy photographs with Congo red staining were used to identify the cellulose fibers if raw herbal powder had been added to the herbal pharmaceutical product. Moreover, water solubility and crude fiber content examination were used to inspect for potential herbal additives to the herbal pharmaceutical products. The results demonstrate that the contents of the herbal ingredients of saikosaponin A, saikosaponin D, ferulic acid, and paeoniflorin were around 0.351 ± 0.017, 0.136 ± 0.010, 0.140 ± 0.005, and 2.281 ± 0.406 mg/g, respectively, for this herbal pharmaceutical product. The physical examination data demonstrate that the raw herbal powder had rough, irregular, lumpy, filamentous, and elongated shapes, as well as strong Congo red staining. In addition, water solubility and crude fiber content were not consistent in the herbal pharmaceutical products.

## 1. Introduction

Decocting and concocting herbal formulas are an important part of traditional Chinese medicine [1]. Based on the ancient process of cooking traditional Chinese medicines, the herbs are placed in water and then cooked in a ceramic or stainless pot, preferably not cast iron or aluminum. The water is brought to a rolling boil and then turned down to a low simmer for 20–30 minutes with the lid on. The extract is collected, with the residue left in the pot, and another equal volume of water is added. This procedure of bringing the mixture to boil is repeated, and then the two decoctions are mixed together for administration. However, this complicated and time-consuming process may not be suitable for current lifestyles. Thus, more convenient pharmaceutical herbal products have been replacing the traditional herbal cooking process. Pharmaceutical herbal products are mainly made by industrial manufacturing methods of decoction, filtration, extraction, concentration, spray or fluid bed granulation, coating, and filling [2]. Consequently, the excipient may dilute the herbal content. Starch, edible gum, lactose, and carboxymethyl cellulose are used for granulation to make herbal pharmaceutical product in the dosage forms of granule, powder, pill, pellet, tablet, capsule, or medicinal liquor.

To increase the content of the herbal ingredient, raw herbal powder has been legally added into herbal pharmaceutical products to improve their herbal content. However, raw herbal powder was made by simply milling and sieving directly from the dry herbs, and these may contain pesticide residues [3], heavy metals [4, 5], bacterial contamination [6–8], and other uncertainties. Therefore, it is important to examine the content of marker compounds and herbal additive in Chinese herbal pharmaceutical products [9, 10].

The Database of National Health Insurance Research in Taiwan has made a survey of the herbal formulation of Jia-Wei-Xiao-Yao-San (JWXYS), which is the most frequently used of the classical traditional Chinese medicinal prescriptions [11]. This herbal prescription is commonly used in clinical applications for postmenopausal women with climacteric symptoms [12, 13], functional dyspepsia [14, 15], mood stabilizer swings, insomnia [16], or breast cancer [17]. The first record of JWXYS is in the traditional herbal text Tai-Ping-Hui-Min-He-Ji-Ju-Fang, as edited by the office of “He Ji Ju” in the Song Dynasty (960-1279).). According to the Chinese Pharmacopoeia (2005), the JWXYS formula includes the following 10 herbal medicines: *Radix Angelicae Sinensis *(root of *Angelica sinensis *(Oliv.) Diels, a perennial herb of the family Umbelliferae, Chinese name: Dang-Gui), *Radix Paeoniae Alba *(root of *Paeonia lactiflora *Pall, a perennial herb of the family Ranunculaceae, Chinese name: Shao-Yao), *Radix Bupleuri *(root of the perennial plant *Bupleurum chinense *DC. or *Bupleurum scorznerifolium* Willd, family Umbelliferae, Chinese name: Chai-Hu), *Poria *(sclerotium of the parasitic plant *Poria cocos *(Schw.) wolf of family Polyporaceae, Chinese name: Fu-Ling), *Rhizoma Atractylodis Macrocephalae *(rhizome of the perennial herbaceous plant *Atractylodes macrocephala *Koidz of family Compositae, Chinese name: Bai-Zhu), *Herba Menthae *(stem and leaf of the perennial herbaceous plant *Mentha haplocalyx* Briq., family Labiatae, Chinese name: Bo-He-Ye), *Radix Glycyrrhizae *(root and rhizome of the perennial herbaceous plant *Glycyrrhiza uralensis *Fisch., family Leguminosae, Chinese name: Zhi-Gan-Cao), *Cortex Moutan Radicis *(root bark of the perennial deciduous shrub (Chinese name: Mu-Dan-Pi),* Fructus Gardeniae *(ripe fruit of the evergreen shrub, *Gardenia jasminoides *Ellis., family Rubiaceae, Chinese name: Shan-Zhi-Zi), and *Rhizoma Zingiberis Recens *(rhizome of the perennial herbaceous plant *Zingiber officinale *Rosc., family Zingiberaceae, Chinese name: Wei-Jiang).

Although the bioactive constituents of JWXYS have not been clarified thoroughly, some of the compounds contained in the ingredient herbs have been tested and have shown to have pharmacological properties. For instance, saikosaponin A and saikosaponin D are the bioactive oleanane saponins in *Radix Bupleuri* (Chai-Hu), and it had been reported that saikosaponins have hepatoprotective, antiinflammatory, and immunomodulatory activities [18–20]. Ferulic acid isolated from the roots of *Angelica sinensis *(Oliv.) Diels has shown antioxidant activity [21]. Paeoniflorin isolated from *Radix Paeoniae Rubra* has indicated inhibition of the fluorescent intensity of intracellular Ca^2+^ and of the activities of mitogen-activated protein kinase [22].

Recently, analytical methods have been reported for use in measuring the herbal ingredients of JWXYS [23, 24], but there has been little research on simultaneously determining the marker constituents of this complex herbal formulation. In the current study we describe a high-performance liquid chromatography tandem mass spectrometry (LC-MS/MS) assay with multiple reaction monitoring (MRM) for the measurement of major biomarker compounds of saikosaponin A, saikosaponin D, ferulic acid, and paeoniflorin in JWXYS. 

As mentioned previously, the Chinese herbal pharmaceutical product JWXYSs is made by various manufacturers, and there are different amounts of various raw herbal powders that are added to the herbal pharmaceutical product. To examine the quality of the various brands of pharmaceutical products, these products should be evaluated by observation of outer appearance, particle size, solubility, and the contents of crude fiber by using the following methods: (1) scanning electron microscope (SEM); (2) light microscopy photographs of Congo red stained; (3) solubility test; and (4) crude fiber analysis.

This study develops multiple chemical and physical methods to evaluate the quality of commercially available herbal pharmaceutical products from various pharmaceutical manufacturers. These methods can provide a standard procedure for conducting quality control of Chinese herbal pharmaceutical products.

## 2. Materials and Methods

### 2.1. Reagents and Materials

Saikosaponin A and paeoniflorin were purchased from ChromaDex. Inc., (Irvine, CA, USA). Saikosaponin D was purchased from Nacalai Tesque, Inc., (Kyoto, Japan). Ferulic acid was obtained from Sigma-Aldrich Chemicals (St. Louis, MO, USA). LC/MS grade acetonitrile was obtained from J. T. Baker. Inc., (Phillipsburg, NJ, USA). Ammonium acetate was obtained from E. Merk (Darmstadt, Germany). Deionized water (Millipore, Bedford, MA, USA) was used throughout the entire experiment. The herbal pharmaceutical products of JWXYS were purchased from five different pharmaceutical manufacturers in Taiwan, for example, Sun Ten Pharmaceutical Co., Ltd. (Taipei, Taiwan), Kaiser Pharmaceutical Co., Ltd. (Tainan, Taiwan), Chuang Song-Zong Pharmaceutical Co., Ltd. (Kaohsiung, Taiwan), Koda Pharmaceutical Co., Ltd. (Taoyung, Taiwan), and Sheng Chang Pharmaceutical Co., Ltd. (Taipei, Taiwan). In presenting the results of analysis, the names of these manufacturers have been coded to preserve commercial confidentiality.

### 2.2. High-Performance Liquid Chromatography Tandem Mass Spectrometry

LC-MS/MS analysis was performed on a Shimadzu LCMS-8030 triple quadrupole mass spectrometer equipped with an electrospray ionization interface and coupled to the UPLC system (Shimadzu, Kyoto, Japan). The instrument settings were optimized as follows: interface voltage, 4.5 kV; desolvation line temperature, 250°C; heat block temperature, 400°C; desolvation gas, nitrogen; desolvation gas flow rate, 3 L/min; drying gas, nitrogen; drying gas flow rate, 17 L/min; collision gas, argon; and collision gas pressure, 230 kPa.

The MS/MS spectrometer operating parameters were performed in multiple reaction monitoring (MRM) mode. The chromatographic separation was accomplished using a Kinetex C18 column (2.1 mm × 100 mm, particle size 2.6 *μ*m, Phenomenex, Torrance, CA, USA). The column temperature was maintained at 35°C. The following gradient program was applied to the analyte with the mobile phase consisting of 10 mM ammonium acetate (solvent A) and acetonitrile (solvent B): 0–7 min: 30–90% B; 7–10 min: 90–90% B; 10-11 min: 90–30% B; and 11–15 min: 30-30% B, v/v. The flow rate was 0.2 mL/min and the injection volume was 10 *μ*L.

### 2.3. Standard Solutions

The stock solutions were prepared by dissolving 1 mg of saikosaponin A, saikosaponin D, ferulic acid, and paeoniflorin into 1 mL of 50% (v/v) acetonitrile to a final concentration of 1.0 mg/mL, respectively. All stock solutions were stored at −20°C before use. A series working standard solutions were prepared by dilutions of the stock solution with 50% (v/v) acetonitrile to obtain the following concentrations: 10, 25, 50, 100, 250, 500, 1000, and 2500 ng/mL. Working solutions for QC samples with three level concentrations of 50, 500, and 1000 ng/mL were prepared in the same manner. All the solutions were kept at 4°C and were brought to room temperature before analysis.

### 2.4. Sample Preparation for Extracts of Herbal Pharmaceutical Powders

Each sample was prepared by 0.1 g Chinese pharmaceutical herbal powder immersed in 25 mL of methanol (4 mg/mL), then extracted in an ultrasonic water bath for 15 min, and centrifuged at 13,000 rpm for 10 min at 4°C. The supernatant was filtered through a 0.22 *μ*m syringe filter. The filtrate was diluted to appropriate concentration and then 10 *μ*L was injected into the LC-MS/MS for analysis.

### 2.5. Quantitative Determination of Marker Compounds

The most intense ion detected was selected for quantitative determination. The relative concentration of marker compounds in sample was determined by using the interpolation method of the calibration curve, and each batch of samples was in compliance with the calibration curve at the same time. Using back calculation, the content of marker compounds in JWXYS was calculated using the following formula. The content of marker compound in JWXYS (mg/g) = [determined concentration (ng/mL)/concentration of sample (4 mg/mL)] × dilution ratio.

### 2.6. Method Validation

The linearity of calibration curves was demonstrated by the good determination of coefficients (*r* ^2^) obtained for the regression line, and all calibration curves were required to have a correlation value of at least 0.995. The limitation of detection (LOD) and quantification (LOQ) for each standard were defined at signal-to-noise ratio (S/N) of 3 and 10, respectively. The intraday variation was determined by analyzing the six replicates on the same day and interday variation was determined for consecutive days. The accuracy was estimated by the nominal concentration (*C*_nom⁡_) and the mean value of the observed concentrations (*C*_obs_) as follows: accuracy (%, bias) = [(*C*_obs_ − *C*_nom⁡_)/*C*_nom⁡_] × 100. The precision, relative standard deviation (RSD), was calculated from the observed concentrations as follows: RSD(%) = (standard  deviation  (SD)/*C*_obs_) × 100. Accuracy and precision values within ±15% were considered acceptable in the experimental concentration range, and LOQ values were less than ±20%.

### 2.7. Physical Examination of Additives for Raw Herbal Powder

#### 2.7.1. Scanning Electron Microscope (SEM)

The outer appearance of the herbal pharmaceutical powders was examined using a scanning electron microscope (JEOL JSM-5300, Jeol Ltd., Tokyo, Japan). The herbal pharmaceutical products of JWXYS were purchased from five different pharmaceutical manufacturers in Taiwan. The crushed herbs of JWXYS were purchased from a Chinese traditional herbal medicine store in Taipei and prepared to raw herbal powder in the National Research Institute of Chinese Medicine, Taipei, Taiwan. These herbs were milled by hammer mill (Hung Chuan RT-04, Taipei, Taiwan) and passed through a 60 mesh sieve. Food-grade corn starch was purchased from Sun Right Co., Ltd. (New Taipei, Taiwan) and passed through a 60 mesh sieve.

For sample preparation, the herbal pharmaceutical powder was dried for 24 hours in an oven (DO45, DENGYNG Instruments Co., Ltd., New Taipei, Taiwan) at 40°C, then placed on an aluminum support with glue or double-sided adhesive tape, and coated with gold using a gold sputter module for 90 seconds in a high vacuum evaporator (Ion Sputter JFC-1200, Jeol Ltd., Tokyo, Japan). The samples were subsequently analysed under a scanning electron microscope. Then raw herbal fine powder and corn starch were examined in the same manner.

#### 2.7.2. Light Microscopy Photographs of Congo Red Stained Samples

Samples were prepared to suspension (3% w/w) on a microslide by adding 1-2 drops of glycerol/20% ethanol (1 : 1) mixed solution, covered with a coverslip that had been cleared of bubbles, and finally stained with 0.1% Congo red. Photographs were acquired by light microscopy (Olympus CKX41, Tokyo, Japan) and camera (Canon PowerShot A620, Tokyo, Japan). Red or pink staining was viewed under microscope magnification of 100 times.

#### 2.7.3. Solubility Test

The estimation of water solubility index was carried out according to previous reports [25, 26], with some modifications. First, distilled water was added to 0.45 g herbal pharmaceutical powder along with 30 g deionized water (1.5%, w/w). This was then vortexed and heated in a circulating water bath (BH-230D, YIN DER Instruments Co., Ltd., New Taipei, Taiwan) to the temperatures of 55, 65, 75, 85, and 95°C for 1 hour each. Then the samples were placed in an ice water bath for cooling to room temperature and centrifuged at 8000 g for 20 minutes. The supernatant was collected and put in dry oven at 105°C; the weight of residue was *W*_1_ and that of the residue precipitate in the centrifuge tube was *W*_2_. Solubility was computed by the following formula, and solubility = (*W*_1_/pharmaceutical herbal powder weight) × 100%.

#### 2.7.4. Crude Fiber Analysis

Crude fiber content was determined by following Method no. 32-10, AACC (2000). The sample of pharmaceutical herbal powder (2 g) was digested by 200 mL solution of boiling 1.25% H_2_SO_4_ for 30 min, washed with hot distilled water, and filtered by using a suction apparatus. The sample was then transferred to a 200 mL solution of 1.25% NaOH and treated in the same manner. The residue was dried in an oven at 100°C for 24 hours to achieve constant weight and then ignited in a muffle furnace (DF202, DENGYNG Instruments Co., Ltd., New Taipei, Taiwan) at 550–600°C for 5-6 hours until grey ash was obtained. This was cooled in desiccators and weighed. The crude fiber (%) was calculated using the following formula: crude fiber (%) = (constant weight of residue − weight of ash) × 100/weight of sample.

### 2.8. Statistical Analysis

Statistical analyses were performed with Version 10.0 (SPSS, Chicago, IL, USA) and SigmaPlot 8.0 software. Data is presented as mean ± standard deviation. Analysis of variance followed by the Student's *t* test or one-way ANOVA comparison adjustment was used, and statistically significant differences were defined as *p* < 0.05.

## 3. Results and Discussion

### 3.1. Optimization of LC-MS/MS Conditions

**Figure 1:**
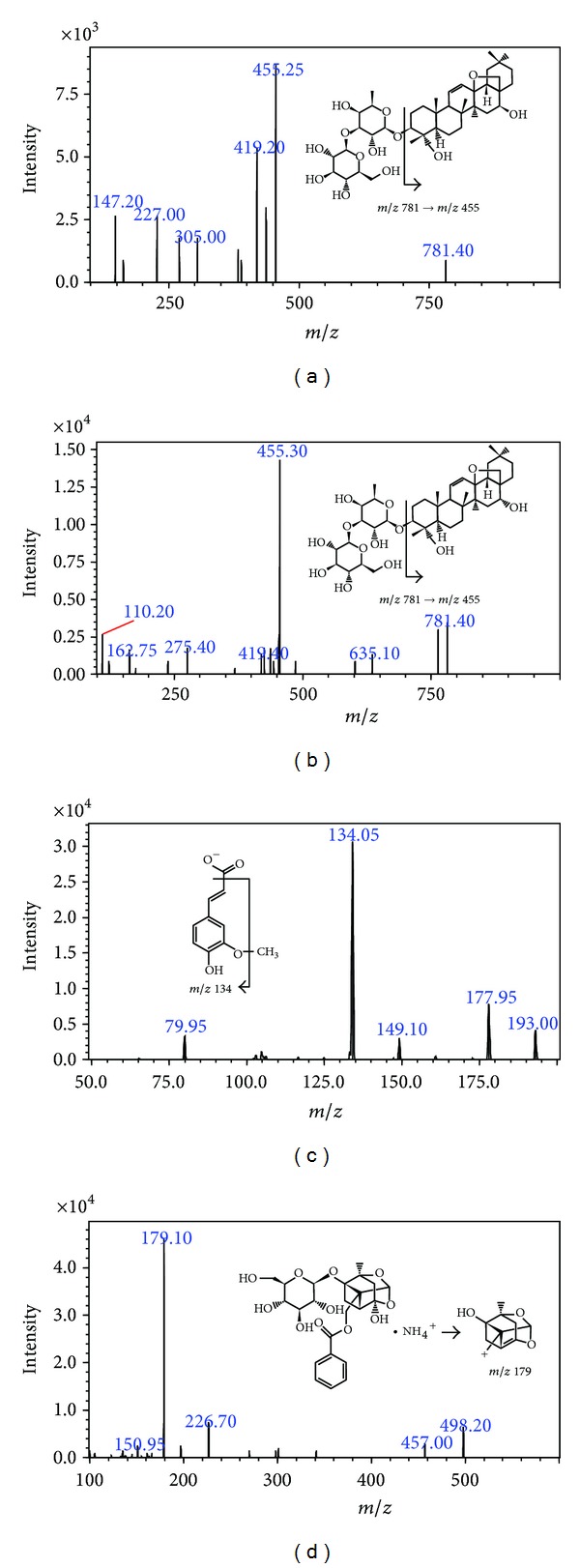
The product ion mass spectra of four marker compounds (a) saikosaponin A; (b) saikosaponin D; (c) ferulic acid; and (d) paeoniflorin.

Identification and determination of the marker ingredients in JWXYS were performed by LC-MS/MS. The ESI-MS spectra of experiment and marker compounds were acquired in both ESI (+) and ESI (−) ionization modes simultaneously. Analytes were quantified by multiple reaction monitoring (MRM) mode performing the following precursor ion (MS 1) to a specific fragment of the product ion (MS 2) pairs of the transitions *m*/*z*: saikosaponin A, *m*/*z* 781.40 [M+H]^+^→*m*/*z* 455.35 (CE −25.0 eV), saikosaponin D, *m*/*z* 781.40 [M+H]^+^→*m*/*z* 455.35 (CE −25.0 eV), ferulic acid, *m*/*z* 193.00 [M−H]^−^→*m*/*z* 134.00 (CE 15.0 eV), and paeoniflorin, *m*/*z* 498.20 [M+NH_4_]^+^→*m*/*z* 179.10 (CE −25.0 eV) (Table 1) (Figure 1).

**Figure 2:**
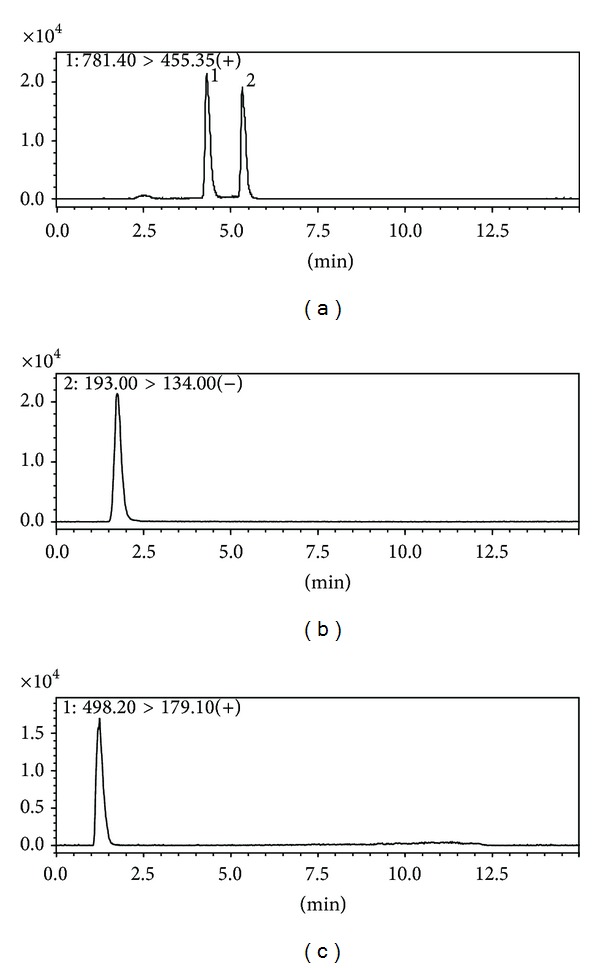
Typical MRM chromatograms of analytes: (a) 1: saikosaponin A, 2: saikosaponin D; (b) ferulic acid; and (c) paeoniflorin.

To optimize the separation of analytes, the gradient elution, proper column, and flow rate were considered pivotal influences on the separation for the analytes. The chromatographic conditions achieved good resolution, appropriate ionization, and coelution. Optimization of LC-MS/MS conditions is required to provide good sensitivity, selectivity, and symmetry for the peaks (Figure 2).

### 3.2. Method Validation

Calibration curves showed good linearity in the range of 25–1000 ng/mL for saikosaponin A, 50–1000 ng/mL for saikosaponin D, 50–1000 ng/mL for ferulic acid, and 50–1000 ng/mL for paeoniflorin, respectively. The calibration curves and correlation coefficients (*r* ^2^) were as follows: *y* = 296.6*x* − 41.67 (*r* ^2^ = 0.998, Saikosaponin A), *y* = 245.1*x* +755.8 (*r* ^2^ = 0.997, Saikosaponin D), *y* = 472.8*x* −8820 (*r* ^2^ = 0.999, Ferulic acid), and *y* = 553.8*x*–1745 (*r* ^2^ = 0.999, paeoniflorin), respectively (Table 2).

The data showed the LOD for the herbal ingredients (Table 2). Peak areas in chromatograms containing the previous lowest concentrations were compared with the signal-to-noise ratio greater than 3 times. Sensitivity was evaluated by the LOQ determinations, which are defined as the lowest concentration that can be reliably and reproducibly measured in at least three replicates. The data showed that the LOQ for saikosaponin A, saikosaponin D, ferulic acid, and paeoniflorin were 25, 50, 50, and 50 ng/mL, respectively.

Precision and accuracy were evaluated by intra- and interday assays. Good linearity was achieved over the calibration range, with all coefficients of correlation greater than 0.995. The RSD values were found to be within the range of 0.11–13.74% for intraday assays and 0.21–10.93% for interday assays, with accuracy ranges of −14.93–6.04 and −4.25–1.92%, respectively. The results are summarized in Table 3, which indicates that precision and accuracy values were within the acceptable range.

### 3.3. Quantitative Determination of the Four Marker Compounds of JWXYS Preparations

In order to investigate the amounts of the marker ingredients in commercially available JWXYS products from various pharmaceutical manufacturers, the most intense ion detected was selected for quantitation analysis. 

The results demonstrate that the contents of saikosaponin A, ferulic acid, and paeoniflorin among samples for the pharmaceutical manufacturers of A–E were situated between 0.116 to 0.351 mg/g, 0.046 to 0.140 mg/g, and 2.128 to 3.497 mg/g, respectively. However, the herbal ingredient of saikosaponin D was detectable only in the samples from pharmaceutical manufacturers B and D. All established contents for the potential active ingredients in samples are shown in Table 4. LC-MS/MS with chemical profiling was provided to rapidly evaluate the chemical consistency among herbal pharmaceutical products of JWXYS.

The composition ratios of this herbal pharmaceutical product from different manufacturers were consistently labeled, but there were different amounts of the four marker ingredients in samples A–E. The levels of the marker compound amounts were influenced by many factors, such as herbal origin, herbal growth time, cultivation period, storage, concoction, decoction method, concentration ratio, granulation process, and product batch. In addition, the extraction from crude herbs and the excipient additives might be different during granulating process [27]. Furthermore, due to the complexity of traditional Chinese herbal medicines, numerous analogues, such as saikosaponin A and saikosaponin D, might be coeluted during analyses. The data show that saikosaponin D was detectable only in samples B and D. This may have been due to differences of herbal origin because *Bupleurum chinense* DC and *B. scorzonerifolium* Willd are both used as authentic herbs for *Bupleuri Radix* (Chinese name: Chai-Hu) in the Chinese Pharmacopoeia [28–30].

In this study, a developed and validated LC-MS/MS method was used to determine saikosaponin A, saikosaponin D, ferulic acid, and paeoniflorin in various brands of Jia-Wei-Xiao-Yao-San simultaneously. According to our results, we will select one of the commercially available Jia-Wei-Xiao-Yao-San to investigate the pharmacokinetics of bioactive components in the near future.

### 3.4. Evaluation of Additives for Raw Herbal Powder

#### 3.4.1. Scanning Electron Microscope (SEM)

**Figure 3:**
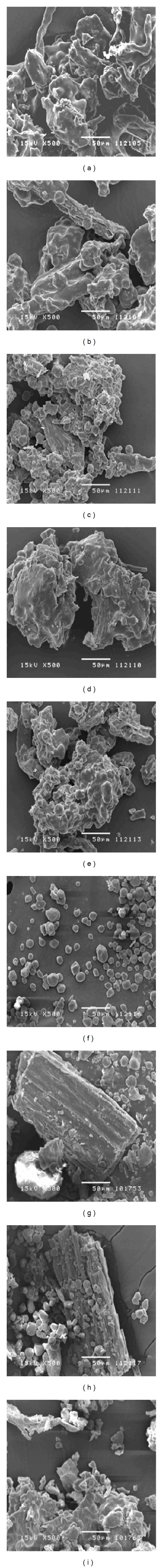
Scanning electron microscope photographs of (a) herbal pharmaceutical product JWXYS of brand A (b) herbal pharmaceutical product JWXYS of brand B, (c) herbal pharmaceutical product JWXYS of brand C, (d) herbal pharmaceutical product JWXYS of brand D, (e) herbal pharmaceutical product JWXYS of brand E, (f) corn starch, (g) raw herbal powder, (h) corn starch: raw herbal powder = 1 : 1, and (i) raw herbal powder: herbal pharmaceutical product JWXYS of brand A = 1 : 1 (×500).

A scanning electron microscope (SEM) was used to assess the possibility of additive raw herbal powder by observing the outer appearance and size of particles. Figures 3(a) to 3(e) show the outer appearance of this herbal pharmaceutical product with gelatinized, irregular shape. In comparison with the herbal pharmaceutical product, it was found that corn starch was granular, uniform, and polygonal in shape (Figure 3(f)) [31], whereas the raw herbal powder was rough, irregular, lumpy, filamentous, and elongated (Figure 3(g)). In general, most plant fibers have uneven, lumpy, or filamentous outer appearances, as shown in the photograph of raw herbal powder (Figure 3(g)). In addition, herbal pharmaceutical products should be uniform or gelatinized due to the manufacturing process of granulation and the addition of starch as an excipient [32]. SEM observations of the outer appearance of the herbal pharmaceutical product, starch, and raw herbal powder could be distinguished clearly.

#### 3.4.2. Light Microscopy Photographs with Congo Red Staining

**Figure 4:**
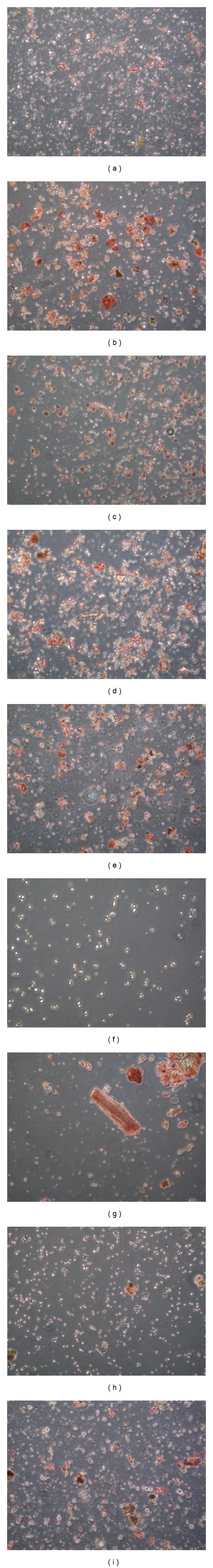
Light microscopy photographs of Congo red stained: (a) herbal pharmaceutical product JWXYS of brand A; (b) herbal pharmaceutical product JWXYS of brand B; (c) herbal pharmaceutical product JWXYS of brand C; (d) herbal pharmaceutical product JWXYS of brand D; (e) herbal pharmaceutical product JWXYS of brand E; (f) corn starch; (g) raw herbal powder; (h) corn starch: raw herbal powder = 1 : 1; and (i) raw herbal powder: herbal pharmaceutical product JWXYS of brand A = 1 : 1 (×100).

The method of identification of the cellulose fibers was assessed by light microscopy photographs with Congo red staining. The photographs show that herbal pharmaceutical products made from different manufacturers (Figures 4(a)–4(e)) and raw herbal powder (Figure 4(g)) were red or pink, but corn starch was not (Figure 4(f)). Congo red has a strong, though apparently noncovalent affinity to polysaccharides (e.g., cellulose fibers), and synthesizes a red complex. It was found that the sample containing fiber components could be clearly observed under the microscope with Congo red staining, and for samples A to E the possibility of having added cellulose fibers could not be ruled out. This method could be a reference index for assessing the possible addition of raw herbal powder to a prepared compound.

#### 3.4.3. Solubility Test

Solubility testing was used to evaluate the water solubility at different temperatures among the herbal samples made by different manufacturers. The results demonstrate that the solubility of herbal pharmaceutical powder for manufactures A–E was 27.88%–46.89% at 55°C, 29.00%–45.17% at 65°C, 29.95%–46.83% at 75°C, 29.95%–47.06% at 85°C, and 32.11%–49.43% at 95°C (Table 5). Theoretically, the amylose, and amylopectin molecules swell or dissolve gradually, and the water solubility of starch may rise with increasing temperature [33, 34]. On the other hand, solubility will decrease as the relative proportion of starch content is decreased by the addition of raw herbal powder. Apparently, there was not a significant increase in the solubility with increasing temperature. Moreover, the results in this study demonstrate that the five samples made by different manufacturers had different solubilities. Thus the differences in starch content of an herbal pharmaceutical product can be evaluated indirectly by the application of solubility test.

#### 3.4.4. Crude Fiber Analysis

The evaluation of addition raw herbal powder was confirmed by crude fiber analysis. Crude fiber content (%) of samples A, B, C, D, and E was 16.601 ± 1.138, 14.815 ± 0.985, 11.618 ± 0.366, 16.336 ± 1.095, and 17.784 ± 0.592%, respectively. Raw herbal powder was 46.798 ± 3.878%, and corn starch was 0.107 ± 0.020%, comparatively. Because raw herbal powder is mainly obtained from herbal plants, there is more cellulose fiber in the raw herbal powder than the compounds. Compared with corn starch, there was almost no crude fiber. The data demonstrate that the crude fiber content for all pharmaceutical products was within the range of corn starch and raw herbal powder, which suggests that these products all contain different proportions of raw herbal powder. The results demonstrate that different proportions of raw herbal powder may be added into herbal pharmaceutical products. Based on the previous analysis, the data from crude fiber analysis can be a rapid and reliable indicator for quality control and determining the addition of raw herbal powder to a compound.

## 4. Conclusion

Chinese herbal prescriptions are complex formulations composed of several herbs and other ingredients, so it is possible that the different characteristics of physical-chemical properties in individual ingredients may affect each other. In this study, a sensitive, rapid, and selective LC-MS/MS method was developed and validated for the simultaneous determination of saikosaponin A, saikosaponin D, ferulic acid, and paeoniflorin in commercially available Jia-Wei-Xiao-Yao-San. In addition, application of several chemical and physical methods to evaluate the quality of a traditional Chinese formulation was established. The methods in this study can provide a standard procedure for quantity control of Chinese herbal pharmaceutical products.

## Figures and Tables

**Table 1:** The analytical conditions of LC-MS/MS for the identification of the four constituents.

Constituents	Molecular weight	RT (min)	Mass fragments		Collision energy (eV)
			Q1 Mass (amu)	Q3 Mass (amu)	
Saikosaponin A	780.98	4.31	781.40 [M + H]^+ ^	455.35	−25.0
Saikosaponin D	780.98	5.33	781.40 [M + H]^+^	455.35	−25.0
Ferulic acid	194.18	1.73	193.00 [M − H]^−^	134.00	15.0
Paeoniflorin	480.46	1.24	498.20 [M + NH_4_]^+^	179.10	−25.0

**Table 2:** Linear ranges, calibration curves, correlation coefficients (*r* ^2^), and detection limits of four constituents using LC-MS/MS.

Constituents	Linear range (ng/mL)	Calibration curve	*r* ^2^	LOD (S/N = 3) (ng/mL)
Saikosaponin A	25–1000	*y* = 296.6*x* − 41.67	0.998	10
Saikosaponin D	50–1000	*y* = 245.1*x* + 755.8	0.997	10
Ferulic acid	50–1000	*y* = 472.8*x* − 8820	0.999	5
Paeoniflorin	50–1000	*y* = 553.8*x* − 1745	0.999	5

**Table 3:** Intraday and interday, precision, and accuracy for the determination of four constituents from the standard samples.

Nominal concentration (ng/mL)	Intraday			Interday		
	Observed concentration (ng/mL)	Precision (%)	Accuracy (%)	Observed concentration (ng/mL)	Precision (%)	Accuracy (%)
Saikosaponin A						
25	24.26 ± 3.33	13.74	−2.98	24.66 ± 1.74	7.04	−1.37
50	48.13 ± 3.25	6.75	−3.73	48.07 ± 2.47	5.14	−3.87
250	252.40 ± 3.24	1.28	0.96	248.14 ± 4.81	1.94	−0.74
500	497.80 ± 13.28	2.67	−0.44	509.28 ± 11.49	2.26	1.86
1000	1000.13 ± 6.46	0.65	0.01	995.71 ± 5.59	0.56	−0.43
Saikosaponin D						
50	44.74 ± 3.88	8.67	−10.51	48.64 ± 2.73	5.60	−2.72
100	98.28 ± 5.57	5.67	−1.72	101.92 ± 4.71	4.62	1.92
250	258.05 ± 6.92	2.68	3.22	247.21 ± 5.25	2.12	−1.11
500	503.66 ± 11.20	2.22	0.73	505.82 ± 3.69	0.73	1.16
1000	996.29 ± 5.47	0.55	−0.37	997.57 ± 2.07	0.21	−0.24
Ferulic acid						
50	42.54 ± 1.28	3.02	−14.93	48.54 ± 5.31	10.93	−2.92
100	100.01 ± 3.38	3.38	0.01	100.95 ± 4.85	4.80	0.95
250	265.09 ± 3.05	1.15	6.04	249.88 ± 10.51	4.21	−0.05
500	493.81 ± 1.86	0.38	−1.24	500.51 ± 12.88	2.57	0.10
1000	999.35 ± 1.12	0.11	−0.06	999.43 ± 6.03	0.60	−0.06
Paeoniflorin						
50	45.52 ± 2.52	5.53	−8.96	47.88 ± 4.17	8.70	−4.25
100	96.55 ± 4.02	4.16	−3.45	101.20 ± 5.53	5.47	1.20
250	261.12 ± 4.89	1.87	4.45	248.78 ± 3.88	1.56	−0.49
500	501.96 ± 4.52	0.90	0.39	503.40 ± 17.55	3.49	0.68
1000	996.61 ± 2.52	0.25	−0.34	998.17 ± 8.86	0.89	−0.18

Notes: data are expressed as mean ± standard deviation (*n* = 6).

Precision: RSD (%) = [standard deviation/*C*_obs_] × 100.

Accuracy: bias (%) = [(*C*_obs_ − *C*_nom⁡_)/*C*_nom⁡_] × 100.

Abbreviation: RSD: relative standard deviation.

**Table 4:** The amounts of saikosaponin A, saikosaponin D, ferulic acid, and paeoniflorin in the herbal pharmaceutical products of JWXYS from different brands.

Brand	Saikosaponin A (mg/g)	Saikosaponin D (mg/g)	Ferulic acid (mg/g)	Paeoniflorin (mg/g)
A	0.126 ± 0.014	ND	0.055 ± 0.004	2.246 ± 0.306
B	0.351 ± 0.017	0.136 ± 0.010	0.140 ± 0.005	2.281 ± 0.406
C	0.271 ± 0.025	ND	0.046 ± 0.001	2.128 ± 0.387
D	0.280 ± 0.037	0.107 ± 0.012	0.057 ± 0.002	3.200 ± 0.568
E	0.116 ± 0.014	ND	0.124 ± 0.011	3.497 ± 0.599

Each value is expressed as mean ± standard deviation (*n* = 3).

ND: not detectable.

**Table 5:** Solubility analysis of herbal pharmaceutical products of JWXYS at different temperatures.

Sample Brand	Solubility (%)				
	55°C	65°C	75°C	85°C	95°C
A	40.65 ± 2.08	40.42 ± 2.78	39.30 ± 1.72	42.39 ± 3.97	44.01 ± 2.36
B	44.16 ± 1.61	43.24 ± 2.06	43.74 ± 0.72	42.81 ± 1.83	44.74 ± 1.04
C	27.88 ± 2.16	29.00 ± 2.49	29.95 ± 2.24	29.95 ± 1.48	32.11 ± 0.12
D	46.89 ± 2.48	45.17 ± 3.08	46.83 ± 1.62	47.06 ± 2.21	49.43 ± 3.21
E	40.03 ± 0.86	40.40 ± 1.49	39.35 ± 2.71	41.11 ± 3.07	42.91 ± 2.67

Each value is expressed as mean ± standard deviation (*n* = 3).
